# Prevalence of polymorphisms of the genes responsible for auto-inflammatory diseases among 236 patients with recurrent fever in a rheumatology institute in Japan

**DOI:** 10.1186/1546-0096-13-S1-P163

**Published:** 2015-09-28

**Authors:** T Miyamae, M Kawamoto, Y Kawaguchi, H Yamanaka

**Affiliations:** 1 Institute of Rheumatology, Tokyo Women's Medical University, Tokyo, Japan

## Introduction

Auto-inflammatory syndromes are defined as conditions caused by an exaggerated innate immune system response, resulting in episodes of spontaneous inflammation affecting multiple organs. The prototypical auto-inflammatory disorders are associated with periodic febrile episodes. *Auto-inflammatory syndromes* now include polygenic diseases, such as Behcet's syndrome and Still's disease; however, the best characterized auto-inflammatory diseases are relatively rare, but florid conditions arising from mutations in single genes. The prevalence of each auto-inflammatory disease varies depending on ethnic background.

## Objectives

To analyze the prevalence of polymorphisms of the genes responsible for auto-inflammatory diseases managed in a single rheumatology institute in Japan.

## Methods

A total of 202 individuals < 40 years of age with recurrent febrile episodes were enrolled in this study. Recurrent fever was defined as > 2 episodes of fever > 38.5 degrees Celsius lasting > 3 days in a year. Infections, autoimmune disorders, and malignancies were excluded as causes of fever prior to enrollment. Genomic DNA was isolated from the patients' peripheral blood and a polymerase chain reaction (PCR) was used to amplify the indicated exons of 10 genes [MEFV (exons 1-10), TNFRSF1A (exons 2-4), MVK (exons 9-11), NLRP3 (exon 3), NOD2 (exon 4), LI1RN (exons 2-4), IL36RN (exons 2-5), PSMB8 (exons 2, 3, and 5), NALP12 (exons 3 and 9), and PSTPIP1 (exons 10 and 11)], which have been reported as the genes responsible for auto-inflammatory diseases. After cleaning the PCR products, cycle sequencing was carried out using the Big Dye^®^ Terminator v3.1 kit and analyzed with an ABI 3130xl Prism Genetic Analyzer. For the most frequently reported 4 genes, genetic polymorphisms within MEFV (exons 1-10), TNFRSF1A (exons 2-4), MVK (exons 9-11), and NLRP3 (exon 3) were examined. With respect to the other 6 genes, the existence of polymorphisms was also determined within NOD2 (from L248R to P727L), LI1RN (from N52KfsX25 to C91F), IL36RN (from R10X to G141Mfs*29), PSMB8 (from T75M to G201V), NALP12 (from T260M to F402L and R1016X), and PSTPIP1 (from A230T to E277D), with reference to the INFEVERS database, an evolving mutation database for auto-inflammatory syndromes (http://fmf.igh.cnrs.fr/ISSAID/infevers/index.php).

## Results

Gene polymorphisms in the targeted genes were identified in 137 of the 236 patients (58.1%) based on INFEVERS. One hundred thirty-five of the 137 (98.5%) were associated with MEFV genes. Other polymorphisms were identified in TNFRSF1A (n=7), NLRP3 (n=5), NOD2 (n=4), MVK (n=2), and PSTPIP1 (n=1).

## Conclusion

Polymorphisms in MEFV were most frequently identified among Japanese patients with recurrent fevers. Further evaluation with clinical features is warranted.

